# Disparities in Syphilis Trends and the Impact of the COVID-19 Pandemic: A Joinpoint Analysis of Florida Surveillance Data (2013-2022)

**DOI:** 10.7759/cureus.69934

**Published:** 2024-09-22

**Authors:** Evan Niu, Rachel Sareli, Paula Eckardt, Candice Sareli, Jianli Niu

**Affiliations:** 1 Office of Human Research, Memorial Healthcare System, Hollywood, USA; 2 Division of Infectious Disease, Memorial Healthcare System, Hollywood, USA

**Keywords:** covid-19, epidemics, joinpoint regression, syphilis, trend analysis

## Abstract

Introduction: Syphilis, a sexually transmitted infection caused by the bacterium ​​​​​​*Treponema pallidum*, has reemerged at an increasing rate globally in recent years, posing a significant public health concern. Visualizing and analyzing time series trends of syphilis across various demographics and geographic regions, and how syphilis trends varied during the pandemic coronavirus disease 2019 (COVID-19), would help public health policymakers develop targeted strategies and interventions to effectively address the rising rates of syphilis, ultimately improving overall public health outcomes.

Methods: A retrospective study was conducted using surveillance data on infectious syphilis cases reported to the Florida Department of Health, between 2013 and 2022. Age-standardized syphilis rates (ASSRs) per 100,000 persons were calculated using the 2000 U.S. standard population data. The average annual percentage change (AAPC) in the ASSRs was analyzed according to age, gender, race, geographic locations, and the COVID-19 pandemic period to quantify the epidemiological syphilis trends using joinpoint regression models.

Results: In Florida, the ASSR significantly increased from 8.65/100,000 in 2013 to 23.08/100,000 in 2022 across different demographic groups, with an overall AAPC of 11.47 (95% confidence interval (CI): 9.85-13.43). This increase in the AAPC was more pronounced in females (AAPC = 20.97; 95% CI, 18.61-24.49) than in males (AAPC = 10.34; 95% CI, 8.19-12.98). The increasing trends of infectious syphilis were observed across all age groups, with greater increases among those aged 25-49 years (AAPC = 12.32; 95% CI, 10.09-15.18), aged 50-69 years (AAPC = 13.42; 95% CI, 9.41-18.89), and aged over 70 years (AAPC = 13.63; 95% CI, 9.23-21.95), compared to those aged less than 24 years (AAPC = 7.86; 95% CI, 7.06-8.81). The increasing trends were comparable across racial groups, with an AAPC of 8.08 (95% CI, 5.47-11.15) for Hispanics, 11.84 (95% CI, 10.02-14.09) for non-Hispanic Whites, 10.49 (95% CI, 8.75-12.66) for non-Hispanic Blacks, and 11.29 (95% CI, 5.28-19.57) for non-Hispanic individuals of other races, respectively. The AAPC for the COVID-19 pandemic period was 12.99 (95% CI, 8.48-16.21), which was comparable to the pre-pandemic period (AAPC = 11.58; 95% CI, 10.17-12.76), indicating that this upward trend in infectious syphilis persisted throughout the COVID-19 pandemic. At the county level, over the 10-year period, the average ASSRs ranged from 0.89/100,000 in 2013 to 25.41/100,000 in 2022, with the estimated AAPCs varied from 2.47 (95% CI, -1.89 to 6.92) in Monroe County to 50.01 (95% CI, 37.71-62.27) in Okaloosa County.

Conclusions: The trend of infectious syphilis increases substantially from 2013 to 2022, with notable differences observed among age, gender, and geographic regions in Florida. Females experienced a greater rise in infectious syphilis cases compared to males. This upward trend in infectious syphilis persisted throughout the COVID-19 pandemic. Efforts are needed to implement strategies to address the rising syphilis rates within high-incidence groups and communities across the state.

## Introduction

Syphilis, an infection caused by *Treponema pallidum*, is a sexually transmitted disease that has created a significant global concern over the past few years, with an estimated 7.1 million new cases in 2020 [1,2]. In the United States, there has been a significant increase in the number of reported syphilis cases, with 207,255 cases reported in 2022, representing an overall increase of 17.3% since 2021 [3]. Men who have sex with men (MSM) are disproportionately affected, accounting for most of all infectious syphilis cases among men [3]. Sexually active individuals are also at higher risk of infection, particularly in communities with elevated rates of sexually transmitted infections (STIs) [3]. The United States Preventive Services Task Force currently recommends screening for syphilis in individuals at higher risk of infection, particularly men who have sex with men, those with HIV or other sexually transmitted diseases, individuals who use illicit drugs, and those with a history of incarceration, sex work, or military service [4].

The rate of syphilis varies based on location and population characteristics [1,5]. People living with human immunodeficiency virus (HIV) are at an increased risk of syphilis infection, especially among MSM and other groups characterized by multiple sexual partners [6,7]. Data from the Centers for Disease Control and Prevention (CDC) revealed a significant rise in the number of syphilis cases among women, which surged by 19.5% in 2022, compared to a 14% increase reported in women in 2018 [8]. A recent systematic review highlighted a rise in the incidence of syphilis among older adults, a demographic that has received comparatively less attention than the younger age incidence [9]. Florida has a large older population and considerable diversity in racial and ethnic composition. It is one of the three states with the highest rates for new HIV diagnoses (15.7 per 100,000 population) in the United States according to the latest CDC reports [3]. Similar to HIV, Florida ranked 17th in the United States for infectious syphilis rates [3]. While the CDC does report national increases in syphilis cases across both genders and all racial/ethnic groups [3], there is a lack of recent analysis of syphilis incidence trends related to demographics in Florida, particularly how syphilis trends shifted during the pandemic of the coronavirus disease 2019 (COVID-19). This highlights the need for real-world data analysis of syphilis cases in the state, which is crucial for developing newer strategies to improve syphilis control and prevention.

In this study, we used syphilis surveillance data published by the Florida Department of Health to analyze the temporal trends and burden of infectious syphilis using joinpoint regression models. The analysis focused on infectious syphilis, which is reported by the Florida Department of Health as primary and secondary stages of syphilis. We examined age, gender, race, and county-level variations in infectious syphilis trends in Florida. We also investigated the impact of the COVID-19 pandemic on infectious syphilis trends by comparing trends before and during the COVID-19 pandemic periods. The results of this study would enhance our knowledge of the burden of infectious syphilis in Florida and assist in developing strategies for effective prevention and control of syphilis. This article was accepted as an oral presentation abstract at the 2024 joint annual meeting of the Infectious Diseases Society of America (IDSA), Society for Healthcare Epidemiology of America (SHEA), the HIV Medicine Association (HIVMA), the Pediatric Infectious Diseases Society (PIDS), and the Society of Infectious Diseases Pharmacists (SIDP) on October 17, 2024.

## Materials and methods

Data sources

Data were extracted from the Florida Department of Health, publicly available at https://www.flhealthcharts.gov/ChartsDashboards/rdPage.aspx?rdReport=STD.DataViewer&cid=0144. The number of infectious syphilis cases and crude rates for each county and each year from 2013 to 2022 were collected and recorded in an Excel spreadsheet. The crude rate was calculated as the number of syphilis cases per 100,000 individuals in that year. Demographic variables included the year of the case’s diagnosis, sex at birth, age at diagnosis, race/ethnicity, and the county of residence. We estimated the crude rates per 100,000 persons for each year by gender, race/ethnicity, and age groups consistent with 0-9 years (infancy and early childhood), 10-24 years (adolescence and young adult), 25-49 years (early middle adulthood), 50-69 years (late middle adulthood), and ≥70 years (late adulthood). The crude rate of syphilis was transformed into an age-standardized syphilis rate (ASSR) per 100,000 persons using the 2000 U.S. standard population data [10]. A 10-year average ASSR was calculated to estimate the infectious syphilis burden between 2013 and 2022.

Statistical analysis

To identify the temporal trends of infectious syphilis cases in Florida, the annual percentage change (APC) in the ASSR and its 95% confidence intervals (CIs) for each trend segment were identified using the Joinpoint Regression Program, Version 5.0 (Statistical Research and Applications Branch, National Cancer Institute). The average annual percentage change (AAPC), a summary measure of the trend of disease incidence over a specified period [11,12], was calculated to demonstrate the yearly change in the ASSR over the 10 years. The joinpoint regression analysis identified significant points where there was a change in a trend, then computed the APC for each segment and the AAPC for the entire period based on the best-fitting model [11]. When no joinpoint was detected, the AAPC was equal to the APC. If the AAPC and its 95% CI were greater than zero (p < 0.05), the ASSR was considered to have an upward trend. If the AAPC and its 95% CI were less than zero (p < 0.05), the ASSR was considered to have a downward trend. Otherwise, the ASSR was considered stable over time. The analysis was stratified by age, sex, racial groups, and county levels, depending on data availability.

To explore the impact of the COVID-19 pandemic on the syphilis trends, we developed a point regression analysis by pre-specifying the joinpoint in 2020, corresponding to the COVID-19 pandemic outbreak. A comparative analysis was performed for the two time periods: the pre-COVID-19 pandemic period (2013−2020) and the pandemic period (2020−2022). Significance testing was two-sided, and a p < 0.05 was considered statistically significant.

Geographical heat maps of Florida were created using a mapping platform in Excel (Microsoft Corporation, Redmond, United States) to qualitatively visualize the county-level spatial distribution of infectious syphilis cases over the state of Florida. These annual county-level ASSRs were averaged over the 10 years, and the average ASSRs were mapped. A heat map of the AAPC over 10 years was also created to demonstrate infectious syphilis trends at the county level qualitatively. Color gradients from light to dark were used to distinguish counties with lower versus higher averaged ASSRs or the AAPCs among the 67 counties.

Ethics approval

This study was reviewed by the Memorial Healthcare System (MHS) institutional review board and was ruled exempt from review because it uses publicly available, deidentified data (MHS.2024.033).

## Results

Syphilis rates in Florida in 2013-2022

**Figure 1:**
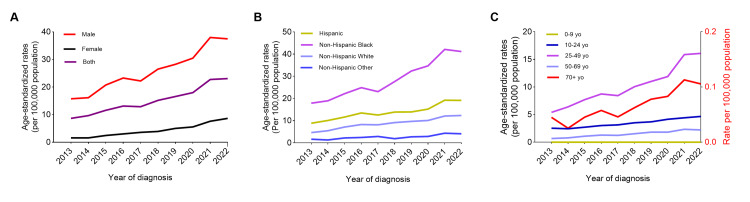
Changes of annual age-standardized syphilis rates in Florida, 2013-2022, stratified by sex, racial, and age groups. The age-standardized syphilis rates in Florida from 2013 to 2022 are depicted by sex (A), racial groups (B), and age groups (C). Age 10-24, 25-49, and 50-69 years are depicted on the left axis of the (C), while age 0-9 and ≥ 70 years are depicted on the right y-axis of the (C).

Figure 1 displays the time series data of the ASSR from 2013 to 2022. Overall, the ASSR increased by 167% (8.65-23.08 per 100,000) from 2013 to 2022 (Figure 1A). All subgroups evaluated showed increasing trends of syphilis from 2013 through 2022, according to sex, race/ethnicity, age, and geographic region. While the ASSR was consistently higher for males than for females from 2013 to 2022, females experienced a much higher increase of 447% (1.58-8.65 per 100,000 women), compared to a 138% increase for males (15.74-37.47 per 100,000 men) (Figure 1A).

The ASSR has increased in all race/ethnicity groups from 2013 to 2022, with Black individuals disproportionately exhibiting a higher ASSR, followed by Hispanic, non-Hispanic White, and non-Hispanic Other races (Figure 1B). There was a 130% increase in the ASSR for Black individuals (17.89-41.19 per 100,000), a 116% increase for Hispanic (8.86-19.15 per 100,000), a 166% increase for non-Hispanic White (4.64-12.36 per 100,000), and a 148% increase for non-Hispanic Other races (1.66-4.11 per 100,000), respectively.

The ASSR showed variations across different age groups (Figure 1C). The infancy and early childhood aged 0-9 years had too few of infectious cases to calculate ASSRs appropriately. Among calculable rates, age groups show a consistent increase, with the highest ASSR in those aged 25-49 years, followed by 10-24 years, 50-69 years, and ≥70 years. Specifically, the ASSR increased by 85% in young adulthood (2.53-4.67 per 100,000), 198% in early middle adulthood (5.4-16.1 per 100,000), 229% in late middle adulthood (0.67-2.21 per 100,000), and 120% in late adulthood (0.05-0.11 per 100,000) individuals from 2013 to 2022.

Trends in the ASSRs identified by joinpoint regression analysis

**Figure 2:**
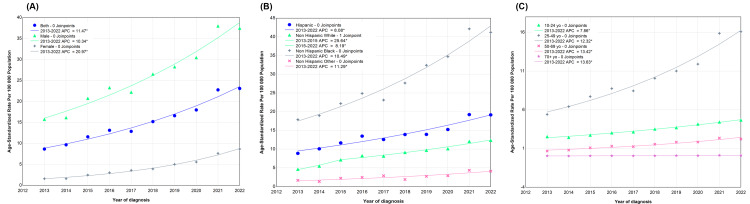
Trends in age-standardized syphilis rates in Florida, 2013-2022, stratified by age, sex, and racial groups. The joinpoint regression models were employed to analyze trends in age-standardized syphilis rates stratified by sex (A), racial/ethnicity group (B), and age group (C). The age group under 9 years (0-9 years) was excluded due to very few infectious cases (1 or even zero) for the given group. APC, annual percentage change. *p < 0.05.

The joinpoint regression models were fitted for each time series dataset according to sex, race/ethnicity, and age groups. The parameters of the regression models displayed in Figure 2 are listed in Table 1. The overall analysis found no joinpoints (Figure 2A), so the APC was equivalent to the AAPC, displaying an increasing trend in the ASSR from 2013 to 2022 (AAPC = 11.47; 95% CI, 9.85-13.43; p < 0.05). The joinpoint analysis, when divided by sex, showed no joinpoints in either males or females (Figure 2A). The AAPC was equal to the APC in both genders, measuring at 10.34 (95% CI, 8.19-12.98) for men and 20.97 (95% CI, 18.61-24.98) for women (Table 1). The AAPC was higher among females than among males, indicating that women experienced a greater increase in the ASSR from 2013 to 2022.

**Table 1:** Trends in age-standardized syphilis rates by age group, sex, and race/ethnicity, Florida, 2013-2022. APC, annual percentage change; AAPC, average annual percentage change; CI, confidence interval; N/A, not applicable. ^a^A model has not been fitted because the data did not meet the modeling criteria. ^b^The linear model with one joinpoint best described the trend. *p < 0.05 for a specific trend.

	Segment 1		Segment 2		2013-2022
	Period	APC (95% CI)	Period	APC (95% CI)	AAPC (95% CI)
Overall	2013-2022	11.47 (9.85-13.43)*	N/A	N/A	11.47 (9.85-13.43)*
Age group					
0-9 yo^a^	2013-2022	N/A	N/A	N/A	N/A
10-24 yo	2013-2022	7.86 (7.06-8.81)*	N/A	N/A	7.86 (7.06-8.81)*
25-49 yo	2013-2022	12.32 (10.09-15.18)*	N/A	N/A	12.32 (10.09-15.18)*
50-69 yo	2013-2022	13.41 (9.41-18.88)*	N/A	N/A	13.41 (9.41-18.88)*
≥70 yo	2013-2022	13.63 (9.23-21.95)*	N/A	N/A	13.63 (9.23-21.95)*
Gender					
Male	2013-2022	10.34 (8.19-12.98)*	N/A	N/A	10.34 (8.19-12.98)*
Female	2013-2022	20.97 (18.61-24.48)*	N/A	N/A	20.97 (18.61-24.48)*
Race/ethnicity					
Hispanic	2013-2022	8.08(5.47-11.15)*	N/A	N/A	8.08(5.47-11.15)*
Non-Hispanic White^b^	2013-2015	25.64 (13.78-38.36)*	2016-2022	8.19 (5.14-9.71)*	11.84 (10.02-14.09)*
Non-Hispanic Black	2013-2022	10.49 (8.75-12.66)*	N/A	N/A	10.49 (8.75-12.66)*
Non-Hispanic Other	2013-2022	11.28 (5.28-19.57)*	N/A	N/A	11.28 (5.28-19.57)*

When separated by racial groups, the analysis revealed zero joinpoint in Hispanic, non-Hispanic Black, or non-Hispanic other races (Figure 2B). The AAPC was equivalent to the APC in Hispanic, non-Hispanic Black, or non-Hispanic Other races, measuring at 8.08 (95% CI, 5.47-11.15), 10.49 (95% CI, 8.75-12.66), and 11.28 (95% CI, 5.28-19.27), respectively (Table 1). Among non-Hispanic Whites, two distinct increasing trends were observed, with an APC of 25.64 (95% CI, 13.78-38.36) from 2013 to 2015, followed by an APC of 8.19 (95% CI, 5.14-9.71) from 2016 to 2022 (Figure 2B), suggesting a decline in the magnitude of the annual increase in infectious syphilis cases from 2016 to 2022. Over the entire period from 2013 to 2022, the AAPC was 11.84 (95% CI, 10.02-14.09), indicating an overall increase in infectious syphilis cases in non-Hispanic White individuals (Table 1). All race/ethnic groups exhibited an upward trend, and the AAPCs were comparable across the various racial groups (Table 1).

Among the age groups, the analysis found no joinpoint in each age group (Figure 2C). The AAPC was equivalent to the APC in individuals aged 10-24 years, 25-49 years, 50-69 years, or ≥70 years, measuring at 7.86 (95% CI, 7.06-8.81), 12.32 (95% CI, 10.09-15.18), 13.41 (95% CI, 9.41-18.88), and 13.63 (95% CI, 9.23-21.95), respectively, indicating that infectious syphilis cases were increasing in these age groups over the 10 years from 2013 to 2022 (Table 1). The rate of annual increase in infectious syphilis cases in those aged 10-24 years was comparatively lower than that observed in other age groups (Figure 2C). Given that the ASSR was zero in those aged 0-9 years, which had much fewer infectious cases to calculate appropriate ASSRs, analysis for this age group was omitted.

Geographic distribution of syphilis cases in Florida counties

**Figure 3:**
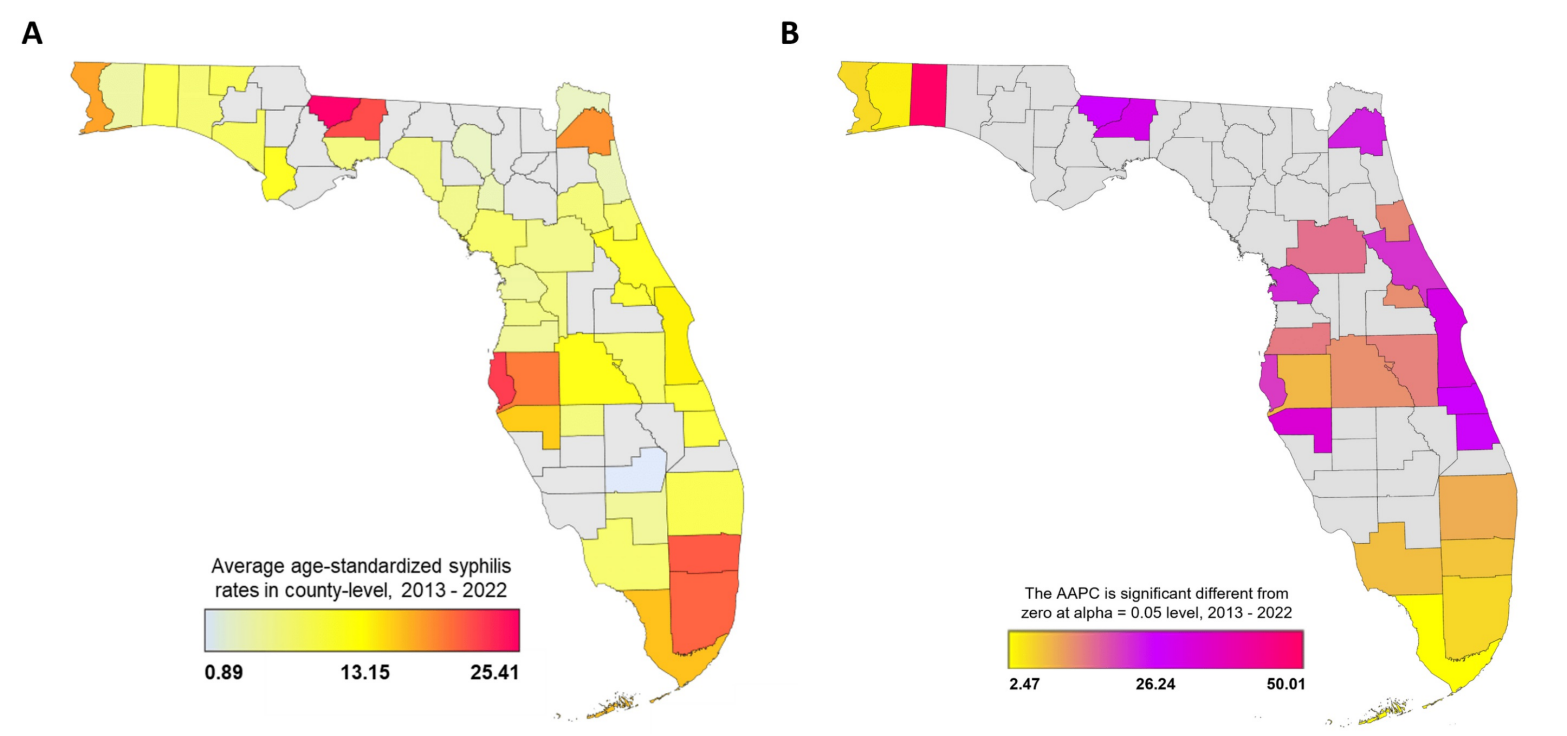
Geographic distribution of infectious syphilis cases in Florida, 2013-2022. The age-standardized syphilis rates at county levels in each year were averaged from 2013 to 2022 and mapped (A). The AAPC across the entire 10-year period by county levels is mapped (B). The Florida map charts were built using Microsoft Excel’s GeoNames software. AAPC, average annual percentage change.

To visualize the distribution of infectious syphilis over the state of Florida, the annual ASSR was averaged at the county level for 2013-2022 and was then mapped (Figure 3A). The average ASSRs ranged from 0.89/100,000 in 2013 to 25.41/100,000 in 2022, as indicated by the color scale ranging from gray to red (Figure 3A). Red hot spots for infectious syphilis cases were observed in Gadsden, Pinellas, Leon, Broward, Miami-Dade, and Orange counties, with ASSRs of 25.41, 22.59, 21.89, 21.25, 20.78, and 20.47 per 100,000, respectively, indicating higher rates in these areas. It should be noted that the county-level ASSRs showed high variability due to very few infectious cases in some counties (Table 2).

**Table 2:** Age-standardized syphilis rates and AAPC at the county level in Florida, 2013-2022. ASSR, age-standardized syphilis rate (per 100,000 population); AAPC, average annual percentage change; CI, confidence interval; N/A, not applicable, data did not meet the modeling criteria.

County	ASSR										Average ASSR (2013-2022)	AAPC (95% CI) (2013-2022)
	2013	2014	2015	2016	2017	2018	2019	2020	2021	2022		
Florida	8.65	9.67	11.57	13.12	12.87	15.19	16.60	17.97	22.76	23.09	15.15	11.47 (9.85, 13.43)
Alachua	7.19	8.32	17.33	34.29	15.79	20.22	20.54	31.22	24.21	20.46	19.96	14.78 (3.77, 25.49)
Baker	0.00	4.00	0.00	4.01	0.00	0.00	11.50	11.11	10.11	11.03	5.18	N/A
Bay	0.00	1.32	2.65	3.03	11.03	5.88	9.43	10.98	27.06	12.82	8.42	N/A
Bradford	0.00	9.07	0.00	16.67	11.85	10.98	3.71	3.61	16.04	0.00	7.19	N/A
Brevard	3.46	3.90	4.33	8.90	10.80	11.61	12.27	19.73	34.47	30.46	13.99	30.33 (22.49, 38.44)
Broward	15.19	18.00	16.19	16.02	17.87	23.98	23.26	25.87	29.49	26.62	21.25	7.89 (4.79, 10.96)
Calhoun	0.00	0.00	0.00	12.45	7.37	4.69	15.47	0.00	4.92	25.85	7.07	N/A
Charlotte	2.12	4.30	0.32	3.22	1.08	2.44	7.24	4.19	10.03	10.13	4.51	26.30 (9.38, 45.84)
Citrus	1.22	3.69	2.48	2.33	2.82	5.21	5.88	6.95	6.92	14.51	5.20	22.43 (14.64, 30.07)
Clay	0.58	1.59	2.21	6.95	7.44	3.00	5.59	4.97	9.32	20.16	6.18	33.52 (9.59, 61.80)
Collier	4.54	7.03	6.53	5.05	6.66	4.96	10.22	5.59	12.61	13.67	7.69	8.52 (3.26, 10.05)
Columbia	1.77	1.62	9.86	3.40	10.26	6.14	13.20	12.77	11.88	13.98	8.49	25.33 (16.36, 34.99)
Miami-Dade	16.20	16.62	18.50	20.26	17.96	20.13	21.24	22.74	26.10	28.03	20.78	6.18 (4.84, 7.19)
DeSoto	0.00	6.37	3.35	2.17	0.00	3.24	3.21	0.00	6.25	23.93	4.85	N/A
Dixie	0.00	7.78	3.64	0.00	7.31	7.53	8.54	0.00	19.73	7.51	6.20	N/A
Duval	4.66	7.37	9.35	11.64	18.38	20.48	24.01	23.78	34.29	33.56	18.75	24.38 (22.49, 26.09)
Escambia	14.77	9.10	24.28	17.24	15.49	15.79	7.63	15.24	28.22	30.59	17.84	5.88 (-5.91, 19.01)
Flagler	11.93	1.46	1.45	5.77	11.09	9.67	3.75	8.39	11.95	12.78	7.83	13.52 (-9.83, 43.05)
Franklin	9.04	0.00	0.00	9.10	0.00	54.09	9.59	14.44	62.18	0.00	15.84	N/A
Gadsden	7.16	7.09	3.77	23.24	31.40	43.19	27.64	36.14	25.96	48.54	25.41	26.54 (4.23, 52.33)
Gilchrist	0.00	0.00	4.00	0.00	0.00	15.67	7.89	5.65	0.00	7.59	4.08	N/A
Glades	0.00	0.00	0.00	0.00	0.00	0.00	8.92	0.00	0.00	0.00	0.89	N/A
Gulf	8.39	0.00	12.15	0.00	6.12	0.00	19.88	23.59	9.59	32.72	11.24	N/A
Hamilton	15.73	8.01	0.00	0.00	23.70	7.98	7.90	16.04	20.28	24.01	12.37	N/A
Hardee	0.00	0.00	8.49	4.28	0.00	0.00	4.41	4.37	28.23	18.15	6.79	N/A
Hendry	2.95	5.92	2.56	2.92	0.00	2.18	10.90	2.10	9.89	15.02	5.44	N/A
Hernando	0.00	3.13	1.56	9.63	6.87	6.27	6.18	8.24	10.54	12.46	6.49	N/A
Highlands	4.69	0.00	5.35	7.75	7.00	0.00	2.23	8.68	13.61	13.74	6.31	N/A
Hillsborough	13.10	16.82	17.27	16.19	13.67	17.43	18.55	23.34	30.86	30.00	19.72	9.08 (4.01, 13.98)
Holmes	0.00	3.51	0.00	5.92	0.00	5.82	21.06	0.00	5.94	45.62	8.79	N/A
Indian River	4.18	2.15	2.48	3.37	9.70	16.92	13.57	11.08	20.17	20.06	10.37	26.60 (5.26, 52.06)
Jackson	0.00	0.00	2.40	1.40	4.63	5.99	17.87	20.22	21.92	45.19	11.96	N/A
Jefferson	8.09	8.14	10.07	0.00	8.40	8.37	12.37	46.01	34.11	42.28	17.78	N/A
Lafayette	0.00	0.00	0.00	0.00	0.00	0.00	0.00	23.82	0.00	0.00	2.38	N/A
Lake	5.39	3.40	6.14	8.98	5.24	7.94	8.48	9.33	10.34	10.21	7.54	10.74 (7.11, 14.18)
Lee	4.72	4.19	4.90	9.73	7.70	6.85	14.32	12.37	21.74	15.87	10.24	19.61 (12.96, 26.04)
Leon	6.68	7.15	7.76	8.68	6.74	22.95	34.04	36.81	41.87	46.25	21.90	29.67 (19.04, 41.53)
Levy	0.00	0.00	3.15	18.48	3.41	3.28	13.30	13.29	9.80	15.25	8.00	N/A
Liberty	0.00	0.00	13.53	0.00	10.20	0.00	0.00	0.00	0.00	12.89	3.66	N/A
Madison	5.96	0.00	0.00	0.00	0.00	5.84	17.39	5.91	12.09	12.44	5.96	N/A
Manatee	1.38	2.17	12.40	18.74	32.26	22.94	23.43	17.78	16.76	11.21	15.91	32.99 (24.52, 42.29)
Marion	1.76	3.63	3.67	3.41	5.53	8.27	8.96	9.57	10.86	7.54	6.32	15.67 (9.76, 23.17)
Martin	1.97	5.27	3.17	1.96	4.94	12.81	5.40	8.96	11.01	14.48	7.00	21.19 (9.80, 33.21)
Monroe	23.12	14.41	10.74	9.34	12.66	8.18	20.51	11.62	26.15	28.18	16.49	2.47 (-1.89, 6.92)
Nassau	0.00	0.00	0.00	0.00	0.00	8.42	4.47	5.11	8.45	8.14	3.46	N/A
Okaloosa	1.18	0.58	2.14	4.44	7.81	10.79	7.40	16.54	22.74	19.39	9.30	50.01 (37.71, 62.27)
Okeechobee	2.96	0.00	2.94	0.00	5.61	0.00	5.63	10.25	7.25	9.16	4.38	N/A
Orange	12.11	14.20	18.59	22.51	18.04	19.83	21.73	23.44	28.26	26.00	20.47	9.37 (5.90, 11.92)
Osceola	4.74	4.73	4.19	7.03	6.14	8.12	7.43	11.83	12.65	11.64	7.85	14.06 (11.26, 16.61)
Palm Beach	7.38	5.31	8.63	7.56	8.94	7.12	8.88	9.30	11.80	15.81	9.07	30.48 (12.29, 43.01)
Pasco	2.52	4.20	4.40	3.83	2.03	3.35	8.72	8.04	7.92	9.86	5.49	14.78 (4.47, 26.19)
Pinellas	6.71	10.05	19.71	22.57	19.35	23.39	25.95	24.80	31.89	41.45	22.59	20.69 (14.97, 26.63)
Polk	5.90	8.86	9.96	9.10	5.96	11.06	13.69	13.77	19.04	20.82	11.82	12.81 (6.56, 19.48)
Putnam	3.51	0.00	0.00	3.83	4.64	9.29	10.56	9.14	7.53	22.61	7.11	N/A
St. Johns	0.60	0.00	2.74	1.08	5.09	7.09	5.43	3.49	7.12	7.44	4.01	N/A
St. Lucie	3.45	3.56	1.55	3.24	11.04	14.85	14.94	11.99	15.32	19.22	9.92	25.73 (7.11, 48.89)
Santa Rosa	2.74	3.86	8.05	7.73	3.05	3.02	3.18	6.65	6.31	4.89	4.95	3.99 (-14.48, 25.97)
Sarasota	4.10	4.49	3.84	9.58	15.05	27.81	12.89	12.46	12.02	13.55	11.58	15.24 (3.11, 28.47)
Seminole	6.44	6.77	7.86	9.07	9.23	7.58	10.17	11.24	18.54	17.84	10.47	12.98 (8.28, 16.22)
Sumter	4.75	1.99	6.80	7.96	4.43	2.16	3.82	12.39	12.21	10.07	6.66	12.59 (-2.38, 30.12)
Suwannee	0.00	2.73	8.21	0.00	2.75	5.73	2.68	5.50	2.99	2.88	3.35	N/A
Taylor	0.00	0.00	0.00	4.92	8.13	11.05	15.99	10.02	0.00	16.72	6.68	N/A
Union	0.00	7.60	12.87	0.00	20.72	25.67	46.93	7.83	6.85	20.16	14.86	N/A
Volusia	4.73	4.47	4.50	7.06	6.25	8.35	14.68	15.87	23.92	23.29	11.31	21.60 (18.61, 26.68)
Wakulla	3.22	0.00	0.00	0.00	0.00	10.24	23.89	10.00	10.28	9.56	6.72	N/A
Walton	3.95	0.00	11.84	5.60	5.43	16.57	5.16	2.52	5.62	17.13	7.38	N/A
Washington	9.08	0.00	0.00	9.15	4.48	4.37	20.54	8.67	13.38	24.74	9.44	N/A

The estimated AAPCs for the county-level ASSRs are displayed in Figure 3B. The AAPCs varied from 2.47 (95% CI, -1.89 to 6.92) in Monroe County to 50.01 (95% CI, 37.71, 62.27) in Okaloosa County, as indicated by the color scale ranging from yellow to red. The gray areas on the map indicate that the AAPCs could not be computed due to much fewer infectious cases in the counties or in those years, giving an ASSR of zero (Table 2). Most counties with a computed AAPC, including Okaloosa, Clay, Manatee, Brevard, Palm Beach, Leon, Indian River, Gadsden, Charlotte, Columbia, St. Lucie, Broward, Collier, Miami-Dade, Escambia, Hillsborough, Monroe, Orange, Osceola, and Santa Rosa, showed positive AAPCs, indicating an increasing trend of infectious syphilis in these counties.

Impact of the COVID-19 pandemic on temporal trends of syphilis

**Figure 4:**
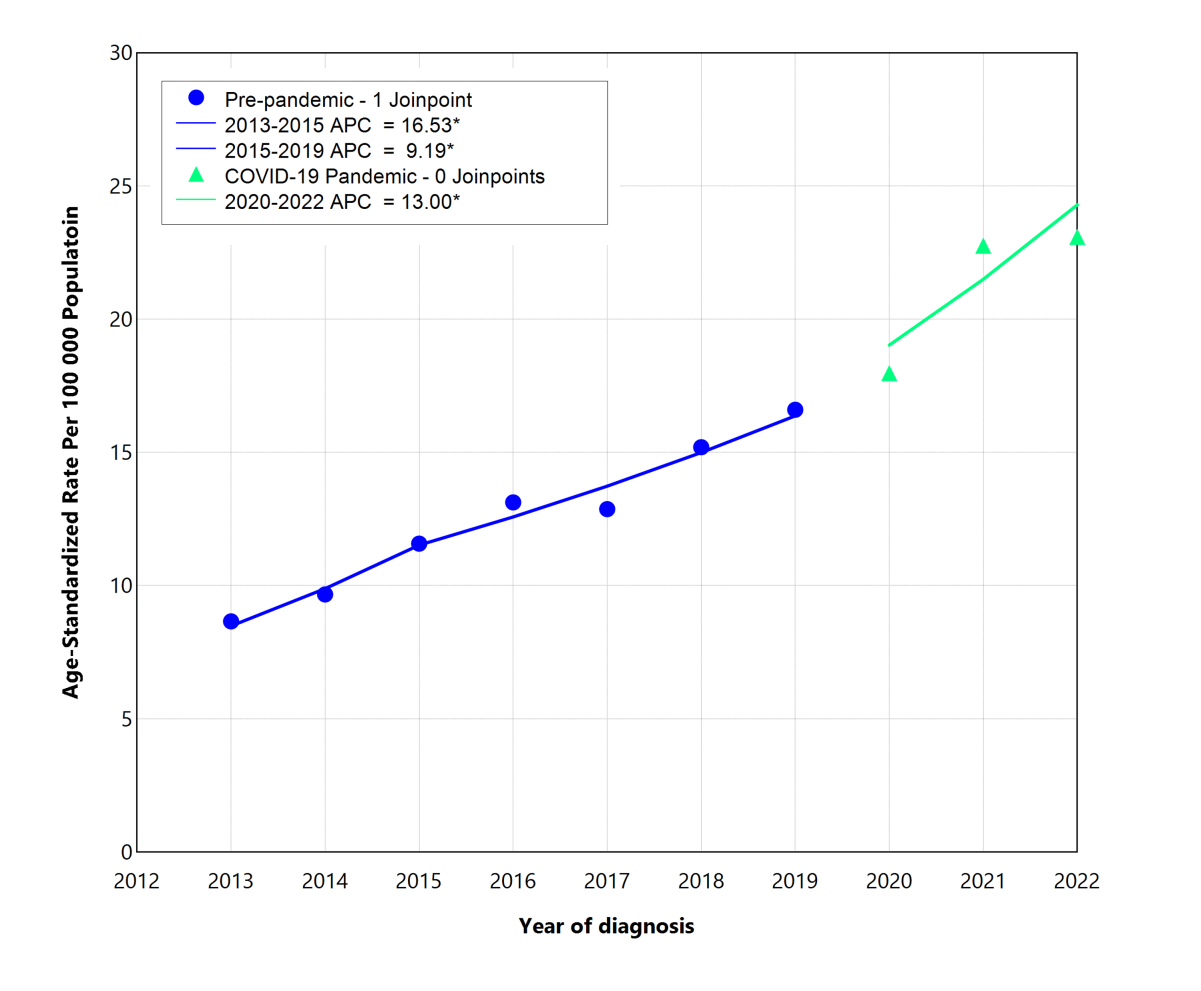
Trends in age-standardized syphilis rates in Florida, before and during the COVID-19 pandemic. The joinpoint regression models were employed to analyze trends in age-standardized syphilis rates for the two time periods: the pre-COVID-19 pandemic period (2013-2019) and the pandemic period (2020-2022). APC, annual percentage change. *p < 0.05.

The infectious syphilis trends impacted by the COVID-19 pandemic are displayed, highlighting the years 2020 to 2022 as the period coinciding with the COVID-19 pandemic (Figure 4). The average ASSR increased from 12.52/100,000 in the pre-COVID-19 years (2013-2019) to 21.27/100,000 during the COVID-19 pandemic years (2020-2022). The joinpoint analysis identified one joinpoint during the pre-pandemic period, with an APC of 16.53 (95% CI, 12.04-21.15) from 2013 to 2015 and an APC of 9.18 (95% CI, 5.12-10.58) from 2016 to 2019. No joinpoint was identified during the pandemic period, which had an APC of 12.99 (95% CI, 8.48-16.21) (Figure 4). The AAPC over the pre-pandemic years (2013-2019) was 11.58 (95% CI, 10.16-12.76), which was comparable to the COVID-19 pandemic period (2020-2022) (AAPC = 12.99; 95% CI, 8.48-16.21), indicating a consistent increasing trend of infectious during the pandemic period (Table 3).

**Table 3:** Trends in age-standardized syphilis rates before and during the COVID-19 pandemic. *p < 0.05; APC, annual percentage change; AAPC, average annual percentage change; CI, confidence interval; N/A, not applicable.

Period	Segment 1		Segment 2		AAPC (95% CI)
	Year	APC (95% CI)	Year	APC (95% CI)	
Pre-pandemic	2013-2015	16.53 (12.04-21.15) *	2015-2019	9.19 (5.12-10.58) *	11.58 (10.16-12.76) *
During the pandemic	2020-2022	12.99 (8.47-16.21) *	N/A	N/A	12.99 (8.47-16.21) *

## Discussion

This study examined the trend of infectious syphilis from 2013 to 2022 in Florida. The ASSR increased significantly from 2013 to 2022. The highest ASSRs were observed among males, non-Hispanic Black individuals, and those aged 25 to 49 years. There has been a consistent upward trend for infectious syphilis across age, sex, and racial groups from 2013 to 2022, mirroring the recently reported trends in syphilis prevalence among the US population in 2022 [3,8]. The upward trend of infectious syphilis persisted through the COVID-19 pandemic within the Florida population. Our findings may significantly contribute to public health by guiding the development of targeted interventions and potentially shaping future policy decisions.

Syphilis affects all age groups, but the highest number of cases occur in the age group of 15-49 years [13,14]. Syphilis is more common among males, with a rate of 16.9 per 100,000 males, compared to the rate of 2.3 per 100,000 females [15]. In 2019, 83% of primary syphilis cases occurred in men, and the number of primary syphilis cases in women has nearly tripled between 2016 and 2022 [3,16]. In our study, Florida infectious syphilis cases were more associated with males. We observed an increase in infectious syphilis cases for both genders, with a greater increase among women than among men. The finding of this increasing trend in females is consistent with recent CDC reports indicating a sharp rise in syphilis cases in women [3,8,16]. Multiple factors such as substance abuse, risky sexual behavior, multiple sexual partners, lack of condom use, exchanging drugs or money for sex, and lack of STI services have been proposed as contributors to syphilis’s rising rates in women [14,16-18]. These findings suggest the urgent need for enhanced access to screening and treatment services in Florida, particularly for females, as the sharp increase in syphilis rate poses a significant risk to both mothers and infants [17].

In addition to gender, racial and ethnic disparities also exist among adults with syphilis. Black people are more likely to become affected by syphilis than White people [14,19]. These disparities have been attributed to the barriers to quality healthcare access in Black populations and differences in socioeconomic conditions [12,20]. This racial disparity with infectious syphilis is represented in Florida data as well. In our study, non-Hispanic Black individuals were disproportionately affected compared to other racial groups, exhibiting the highest rates, averaging 28.5 per 100,000. These findings can help identify population groups requiring targeted public health interventions, inform resource planning, and implement universal screening initiatives within the most disproportionately affected populations. Several key interventions, such as training providers to recognize and mitigate racism within the healthcare system, expanding access to healthcare for underserved populations, continuing educational campaigns, and developing tailored interventions (e.g., community-based screening initiatives) for different age groups and communities, will help address disparities in syphilis rates. The improvements in timely testing, diagnosis, and appropriate treatment of syphilis through tailored strategies can make a significant difference in tackling the upward trend in syphilis cases.

We also evaluated the county-level rates of infectious syphilis in Florida. The Gadsden, Pinellas, Leon, Broward, Miami-Dade, and Orange counties had higher average syphilis rates over the 10 years. Of the 35 counties assessed using joinpoint models, 30 (86%) counties exhibited an increasing trend of infectious syphilis, and five (14%) counties demonstrated a stable trend over the 10 years. Of the nationally reported cases, Florida ranked 17th in the United States for infectious syphilis rates, nearly 15% higher than the national average [3]. These between-county findings provide valuable insights for informing public health decisions, particularly in guiding syphilis screening efforts across different regions.

Starting in March 2020, Florida implemented social distancing and lockdown measures to mitigate the spread of COVID-19, which could theoretically have reduced the transmission of sexually transmitted infections (STIs). In our study, the results indicated a persistent upward trend of the ASSR through the COVID-19 pandemic period. Our findings are consistent with recent reports showing a dramatic rise in syphilis cases during the COVID-19 pandemic [21,22]. Several factors, including unsafe sexual activities, reduced STI screening, and the disruptions of STI programs and services during the pandemic, have been suggested to cause the increase in infectious syphilis cases [23-25]. During the COVID-19 pandemic, healthcare systems may become overwhelmed, shifting focus to urgent care and limiting resources for routine screenings and preventive services. This reduction in access to regular healthcare could result in fewer opportunities for early detection, diagnosis, and treatment of syphilis, contributing to the spread of the infection [21,25]. Additionally, syphilis control programs may have been paused due to the COVID-19 pandemic [21]. As a result, missed opportunities for timely testing and intervention could lead to increased syphilis transmission, particularly among high-risk populations both nationally and in Florida.

This study has several limitations. First, the trend analysis on infectious syphilis cases is based on public health surveillance data with inherent limitations as not all cases may have been reported to public health authorities, leading to an underestimation of the rates. These underreporting cases could impact existing disparities in syphilis rates across different populations and regions. Second, this study does not contain a comprehensive analysis, as the surveillance data may lack key information such as demographic details, clinical characteristics, or risk factors. We were unable to identify factors contributing to the increasing trend in infectious syphilis over time, and the presence of unmeasured confounding factors could not be ruled out. Third, given the statistical model used, trend analyses could not be conducted for countries with low syphilis cases in any given year or for the age group 0-9 years, as the minimum number of cases required to fit the joinpoint regression models was not met. It is important to recognize that neonatal infectious syphilis, even at these low case numbers, remains a serious medical and public health problem [26]. In addition, the relatively shorter study period of the COVID-19 pandemic, particularly in the analysis of two segments, may have resulted in very wide confidence intervals. Future prospective studies in large, diverse populations are recommended to monitor syphilis incidence and prevalence over time. Such studies would provide the most accurate representation of the infectious syphilis epidemic in Florida.

## Conclusions

Syphilis rates are rising rapidly worldwide, and Florida ranks 17th in the United States, with rates nearly 15% higher than the national average. This study analyzed a 10-year Florida surveillance dataset, showing a significant upward trend in infectious syphilis from 2013 to 2022. The incidence rates of infectious syphilis increased across all demographic groups, with the highest rates among individuals aged 25-49 years, males, non-Hispanic Blacks, and urban counties. Women experienced the greatest increase in infectious syphilis cases from 2013 to 2022. Syphilis incidence also increased steadily during the COVID-19 pandemic. These findings may significantly contribute to public health by guiding the development of targeted interventions and potentially shaping future policy decisions.
